# PARP-1 and YY1 Are Important Novel Regulators of CXCL12 Gene Transcription in Rat Pancreatic Beta Cells

**DOI:** 10.1371/journal.pone.0059679

**Published:** 2013-03-26

**Authors:** Jelena Marković, Nevena Grdović, Svetlana Dinić, Teodora Karan-Djurašević, Aleksandra Uskoković, Jelena Arambašić, Mirjana Mihailović, Sonja Pavlović, Goran Poznanović, Melita Vidaković

**Affiliations:** 1 Department of Molecular Biology, Institute for Biological Research, University of Belgrade, Belgrade, Serbia; 2 Laboratory for Molecular Hematology, Institute of Molecular Genetics and Genetic Engineering, University of Belgrade, Belgrade, Serbia; University of Hong Kong, Hong Kong

## Abstract

Despite significant progress, the molecular mechanisms responsible for pancreatic beta cell depletion and development of diabetes remain poorly defined. At present, there is no preventive measure against diabetes. The positive impact of CXCL12 expression on the pancreatic beta cell prosurvival phenotype initiated this study. Our aim was to provide novel insight into the regulation of rat CXCL12 gene (*Cxcl12)* transcription. The roles of poly(ADP-ribose) polymerase-1 (PARP-1) and transcription factor Yin Yang 1 (YY1) in *Cxcl12* transcription were studied by examining their *in vitro* and *in vivo* binding affinities for the *Cxcl12* promoter in a pancreatic beta cell line by the electrophoretic mobility shift assay and chromatin immunoprecipitation. The regulatory activities of PARP-1 and YY1 were assessed in transfection experiments using a reporter vector with a *Cxcl12* promoter sequence driving luciferase gene expression. Experimental evidence for PARP-1 and YY1 revealed their *trans*-acting potential, wherein PARP-1 displayed an inhibitory, and YY1 a strong activating effect on *Cxcl12* transcription. Streptozotocin (STZ)-induced general toxicity in pancreatic beta cells was followed by changes in *Cxcl12* promoter regulation. PARP-1 binding to the *Cxcl12* promoter during basal and in STZ-compromised conditions led us to conclude that PARP-1 regulates constitutive *Cxcl12* expression. During the early stage of oxidative stress, YY1 exhibited less affinity toward the *Cxcl12* promoter while PARP-1 displayed strong binding. These interactions were accompanied by *Cxcl12* downregulation. In the later stages of oxidative stress and intensive pancreatic beta cell injury, YY1 was highly expressed and firmly bound to *Cxcl12* promoter in contrast to PARP-1. These interactions resulted in higher *Cxcl12* expression. The observed ability of PARP-1 to downregulate, and of YY1 to upregulate *Cxcl12* promoter activity anticipates corresponding effects in the natural context where the functional interplay of these proteins could finely balance *Cxcl12* transcription.

## Introduction

Type 1 diabetes (T1D) is a multifactorial disease believed to be of immunological origin, precipitated by infections and environmental factors in genetically predisposed individuals. The hallmark of T1D is selective death of pancreatic insulin-producing beta cells resulting from attack by mononuclear cells. The maintenance of an appropriate number of pancreatic beta cells remains a viable interventive measure in diabetes. Detection of novel beta cell growth factors will provide crucial information for strategies that could compensate for depletion and defects of beta cell functioning.

The chemokine (C-X-C motif) ligand 12 (CXCL12) or stromal cell-derived factor-1 (SDF-1) belongs to the CXC group of chemokines. CXCL12 was discovered as a pre-B cell growth-stimulating factor [1], [2]. The CXCL12 is a ligand of two transmembrane receptors, chemokine (C-X-C motif) receptor 4 (CXCR4) and chemokine (C-X-C motif) receptor 7 (CXCR7) [3], [4]. An antidiabetogenic potential of CXCL12 was recently revealed *in vitro* and *in vivo*. Transgenic mice that overexpress CXCL12 in their beta cells are resistant to apoptosis and diabetes. It was shown that CXCL12 stimulates pancreatic beta cell survival by preventing apoptosis via activation of the prosurvival kinase Akt and the resulting upregulation of antiapoptotic protein Bcl-2 and phosphorylation of the proapoptotic protein Bad [5]. Also, beta cell injury induces CXCL12 expression, and the secreted CXCL12 causes the dedifferentiation of adjacent alpha cells into pro-alpha cells. This is an initial step in transdifferentiation of alpha to beta cells [6], [7]. The process of transdifferentiation in the pancreas is of particular interest, since T1D results from an insufficient number of functional beta cells. Furthermore, the human gene for CXCL12 is located on chromosome 10q11.1, near to the T1D susceptibility locus IDDM10, indicating that CXCL12 gene variants could contribute to diabetes development. Analysis of single nucleotide polymorphisms in the CXCL12 gene revealed that the CXCL12-3′A variant is associated with the early onset of T1D in some populations [8], [9].

Since, CXCL12 is proven to be important in pancreatic islet survival, we aimed to advance knowledge concerning the regulation of rat CXCL12 gene (*Cxcl12*) transcription, and for the first time we focused on two transcription factors; the poly(ADP-ribose) polymerase-1 (PARP-1) and the ubiquitous transcription factor Yin Yang 1 (YY1).

PARP-1 is a multifunctional nuclear enzyme involved in the regulation of a variety of nuclear processes, including cell death [10], replication and differentiation [11], telomere activity [12], energy balance for cellular processes [13] and transcription [14]. Aside from binding to DNA breaks [15], PARP-1 binds to specific DNA sequences, thereby regulating transcription of its own [16], [17] and other genes such as *Mcat1* [18], *Pax-6* [19], *MHC II* [20], *Cxcl1* [21], *Reg* [22] and *Bcl-6* [23]. The enzymatic activation of PARP-1 implies the transfer of ADP-ribose moieties to acceptor proteins in the nucleus (heteromodification), including itself (automodification) [24]. With regard to diabetes, PARP-1 deficiency provides protection from experimentally induced diabetes. Namely, PARP-1 knockout (PARP−/−) mice were shown to be resistant to streptozotocin (STZ) -induced diabetes, restoring normal blood glucose and pancreatic islet structure [25]. Moreover, PARP-1 inhibition protects against autoimmune beta cell destruction in NOD mice via induction of apoptosis of islet-infiltrating leukocytes [26].YY1 is a ubiquitous zinc finger transcription factor that can initiate, activate or repress transcription [27]. When the DNA binding motif of YY1 occurs downstream from the transcription start site, it often overlaps with the Kozak sequence [28]. YY1 is originally identified as a DNA-binding nuclear matrix protein with the ability to bind DNA sequences possessing an unwinding propensity [29], [30]. Subsequent transient poly(ADP-ribosyl)ation of YY1 reduces its DNA binding affinity [31]. Functional relations between YY1 and PARP-1 are also relevant in cases where enzymatic PARP-1 activity modulates transcription [32]. YY1 is involved in the regulation of several genes responsible for cellular functions governing cellular stability [33]. YY1 participates in the regulation of *Parp-1* [34], the chemokine receptor *Cxcr4* [35] and the antiinflamatory cytokine *IL-4* [36] that protects against diabetes development [37].

The aim of our study was to investigate the molecular mechanisms that regulate gene transcription of CXCL12, a potential beta cell growth factor. Our results revealed two novel regulators of the CXCL12 gene and elucidated their influence on *Cxcl12* transcription. Furthermore, our investigation clarified *Cxcl12* promoter regulation in the basal state and during STZ-induced pancreatic beta cell injury.

## Materials and Methods

### Bioinformatics

The rat *Cxcl12* promoter sequence was predicted by Genomatix Software GmbH (Munich, Germany). Putative binding sites for YY1 and Sp1 were identified by ALGGEN-PROMO (http://alggen.lsi.upc.es/cgi-bin/promo_v3/promo/promoinit.cgi?dirDB=TF_8.3) and MatInspector (www.genomatix.de).

### Cell Culture and Treatment

The rat pancreatic insulinoma cell line (Rin-5F) (ATCC-CRL-2058) and a generated Rin-5F with a stably integrated human gene for CXCL12 (clone #1) were cultivated in RPMI medium supplemented with 10% FBS and penicillin/streptomycin. NIH3T3 mouse embryonic fibroblasts (PARP-1^+/+^) (ATCC-CRL-1658) and PARP-1 knock-out (PARP-1^−/−^) mouse embryonic fibroblasts were cultivated in DMEM medium supplemented with 10% fetal calf serum and penicillin/streptomycin. Cell culture reagents were obtained from PAA Laboratories GmbH. Rin-5F wt and clone #1 cells were treated with 5 mM STZ (Sigma), established to correspond to EC_50_. In some experiments, wt cells were pretreated with increasing 3-aminobenzamidine (3AB) (Sigma) concentrations, followed by 5 mM STZ for 24 h.

### Cell Viability Assay

Rin-5F wt and clone #1 cell viability was estimated by the 3-(4,5-Dimethylthiazol-2-yl)-2,5-diphenyl tetrazolium bromide (MTT) viability assay. Cells were cultured in a 96-well plate and treated with increasing concentrations of STZ (0.1–15 mM) for 24 h. After removing the medium, 200 µl of MTT (Sigma, M5655) at a concentration of 0.5 mg/ml in RPMI was added to each well. Cells were incubated for 2 h in the dark and the resultant formazan crystals were dissolved in dimethyl sulfoxide. The absorbance was measured at 570 nm. Cell viability was expressed in percentages after comparison with control cells that were assumed to be 100% viable.

### Comet Assay

The levels of DNA damage after increasing times of STZ treatment were estimated by the alkaline Comet assay according to Singh *et al.* [38]. Rin-5F wt cells were grown in 6-well plates and exposed to 5 mM STZ (Sigma) for increasing times (0.5, 1, 3 and 6 h). Control and STZ-treated cells were collected in PBS. The collected cells (10 µl) were mixed with low-melting agarose (0.75%) and applied to a microscope slide. Cells were lysed for 2 h at 4°C in lysis buffer (2.5 M NaCl, 100 mM EDTA, 10 mM Tris, pH 10, 1% Triton X-100). After lysis, microscope slides were incubated for 30 min at 4°C in electrophoresis buffer (300 mM NaOH, 1 mM EDTA, pH 13.0) in order to denature DNA. Damaged DNA fragments were separated at 10 V for 30 min at 4°C. The slides were washed in neutralization buffer (0.4 M Tris–HCl, pH 7.4) and stained with SYBR Green I (1∶10,000 dilution; SYBR; Sigma-Aldrich). Images were analysed with TriTekCometScoree Freeware version 1.5 (http://www.AutoComet.com).

### Preparation of Protein Fractions

Cell protein fractions were prepared with the ProteoJET Mammalian Cell Lysis Reagent and ProteoJET Cytoplasmic and Nuclear Protein Extraction Kit (Fermentas).

### Electrophoretic Mobility-shift Assay (EMSA)

Rat genomic DNA was extracted from the RIN-5F wt cell line. The *Cxcl12* promoter (739 bp) was amplified using biotinylated PCR primers: upstream 5‘-biotin-CAGCACAGCCCTACGTTAGA-3′ and downstream 5‘-biotin-ACAGAGCTGCGAGCCTTGCC-3′. The PCR products were purified using QIAquick Gel Extraction Kit (Qiagen). EMSA was performed in a binding buffer containing 6.25 mM MgCl_2,_ 10% glycerol, 2.5 mM EDTA, 2.5 mM DTT, 250 mM NaCl and 50 mM Tris-HCl (pH 7.5). The nuclear lysate (20 µg) was incubated with binding buffer for 15 min at room temperature. Subsequently, 100 ng of biotinylated DNA fragments were added and incubation was carried out at 37°C for 30 min. Poly(dIdC) (1 µg) was used as a competitor DNA in each binding reaction. For supershift experiments, 1 µg of anti-PARP antibodies (H-250-Santa Cruz; R&D and C2-10-ALEXIS Biochemicals), and 1 µg of anti-YY1 antibody (H-414-Santa Cruz) were added to the protein mixture and incubated at 37°C for 30 min. EMSA was also performed using recombinant PARP-1. The binding reaction containing the binding buffer, 100 ng of biotinylated *Cxcl12* promoter, 100 ng of recombinant PARP-1 (ALEXIS Biochemicals) and 1 µg of poly(dIdC) was incubated for 30 min at 37°C. Reaction mixtures were separated by non-denaturing electrophoresis on a 1% agarose gel at 80 V for 2–3 h in 1×Tris–borate–EDTA buffer (1×TBE) at room temperature. The biotinylated DNA ends conjugated with streptavidin-alkaline phosphatase prevent PARP-1 binding to the DNA ends and allows post-EMSA DNA visualization by the DuoLux chemiluminescent substrate (UltraSNAP detection system; Vector Laboratories) according to the manufacturer's instruction.

### Chromatin Immunoprecipitation (ChIP)

Rin-5F wt cells were treated with 5 mM STZ for 0.5 and 6 h. Control and STZ-treated cells were fixed with 1% formaldehyde (Lach-ner) for 10 min at room temperature, according to the ChIP-IT Express protocol (ActiveMotif). Chromatin was sheared on ice with 20 pulses. Each pulse consisted of sonication for 20 s, followed by a 30 s rest on ice. Sheared chromatin yielded a 200–500 bp smear. Immunoprecipitation was performed using 3 µg of the following antibodies: two different anti-PARP-1 antibodies (Roche and H-250-Santa Cruz), anti-YY1 antibody; (H-414-Santa Cruz), and anti-Sp1 antibody (E-3-Santa Cruz). The cross-link was reversed by heating the samples in Reverse Cross-linking Buffer at 95°C for 15 min, followed by incubation with 1 µg of Proteinase K for 1 h at 37°C. After adding Proteinase K Stop Solution, DNA samples were amplified with specific primers flanking different fragments within the *Cxcl12* promoter. Primer compositions were: upstream 5′-CAGCACAGCCCTACGTTAGA-3′ and downstream 5′-AGAGGCGAAACTGTGTTCCA-3′ for fragment 1; upstream 5′-TGGAACACAGTTTCGCCTCT-3′ and downstream 5′-AAGGGGCGTGTCTGAAGTGT-3′ for fragment 2; upstream 5′-ACACTTCAGACACGCCCCTT-3′ and downstream 5′-ACAGAGCTGCGAGCCTTGCC-3′ for fragment 3.

### Immunoblot Analysis

Samples (20 µg) of proteins separated by SDS-PAGE (12% acrylamide gel) were electroblotted onto a polyvinylidene difluoride (PVDF) membrane. The membranes were blocked for 1 h at room temperature with 5% non-fat dry milk in blotto base buffer (0.1% Tween 20, 20 mM Tris–HCl pH 7.6, 137 mM NaCl). Immunoblot analysis was performed using the following antibodies: anti- PARP-1 (H-250-Santa Cruz), anti-YY1 (H-414-Santa Cruz), anti-SDF-1 (FL-93-Santa Cruz), anti-caspase-3 (H-277-Santa Cruz), anti-PAR (H10-ALEXIS Biochemicals) and anti-β-actin (Abcam, ab8227). Blots were probed by horseradish peroxidase-conjugated secondary antibody. Staining was performed by the chemiluminescent technique according to the manufacturer's instructions (Amersham Pharmacia Biotech).

### Reporter Gene Constructs

Rat genomic DNA extracted from Rin-5F wt cells was PCR-amplified following standard procedures. To amplify the *Cxcl12* promoter we used the following primers: upstream 5′-GGTCGATACTAGTTTGTAAAGACACCAATGACC-3′ and downstream 5′-CCTAAGCCTCGAGTGGGCGGGAGGGCGCGCCGGAGGCT-3′. The amplified *Cxcl12* promoter was cloned in the pMDICluc construct using restriction enzymes SpeI and XhoI. In the generated pCXCL12luc construct, the *Cxcl12* promoter drives the firefly luciferase gene. The ampicillin gene served as a selection marker. The resulting pCXCL12luc construct was sequenced in both directions using the BigDye Terminator v3.1 Cycle Sequencing Kit (Applied Biosystems).

### Transient Transfection

The day before transfection, cells were plated in 24-well plates, being 70% confluent after 24 h. Rin-5F (wt and clone #1) and NIH3T3 (PARP-1^+/+^ and PARP-1^−/−^) cells were transfected with pCXCL12luc and pMDICluc constructs. pMDICluc is a control construct in which the firefly luciferase gene is under the control of the CMV promoter. PARP-1^+/+^ and PARP^−/−^ fibroblasts were cotransfected with both pCXCL12luc and the PARP-1 expression construct pECV PARP. PARP-1^+/+^ fibroblasts were cotransfected with both pCXCL12luc and the pcDNA3.1FLAGYY1 plasmid containing the FLAG-tagged human YY1 coding sequence. In each transfection reaction the *Renilla* luciferase-construct, served as a normalizing transfection control for firefly luciferase. Transfection experiments were performed using Lipofectamine™ 2000 (Invitrogen), according to the manufacturer’s instructions. Plasmid DNA (0.5 µg) and the *Renilla* luciferase-construct (0.065 µg) were diluted in cell culture medium. Lipofectamine™ 2000 (2 µl) was dissolved in the medium and incubated at room temperature for 5 min. The diluted DNA and cationic lipid were combined and incubated for 20 min. Cell culture medium (without antibiotics) supplemented with the plasmid–lipid complexes was added to each well. After 5 h incubation, the medium was replaced by complete medium and incubation was continued for 24 h. The ratio of the luciferase activity units obtained for each cell line transfected with pCXCL12luc and pMDICluc was normalized by dividing the firefly signal by the *Renilla* signal. The activity of the *Cxcl12* promoter was expressed relatively to activity of control CMV promoter.

### Dual-luciferase Reporter Assay System

Luciferase activity was measured with the Dual-Luciferase® Reporter Assay System according to the manufacturer’s recommendations (Promega Corporation). Cells were lysed 24 h after transfection and firefly luciferase activity was measured immediately after adding LAR II reagent. Afterwards, Stop & Glo® reagent was introduced and *Renilla* luciferase activity was recorded.

### RNA Isolation and Real-time RT-PCR (RT-qPCR)

Rin-5F wt cells were exposed to 5 mM STZ for increasing times (0.5, 1, 3 and 6 h). In some experiments cells were pretreated with 50 µM 3AB for 30 min, followed by 6 h STZ treatment. Total RNA from Rin-5F control (wt and clone #1) and STZ-treated wt cells was extracted using the GeneJET RNA Purification Kit (Thermo Fisher Scientific). For cDNA synthesis, 1 µg of the total RNA was treated with DNAse I and reverse-transcribed with RevertAid First Strand cDNA Synthesis Kit (Fermentas) using oligo(dT) primers. For RT-qPCR the Maxima SYBR Green/ROX qPCR Master Mix (Fermentas) was used. mRNA levels were quantitatively determined with an ABI Prism 7000 Sequence Detection system (Applied Biosystems). The fragments were amplified using the following primer sets: upstream 5′-GATTCTTTGAGAGCCATGTC-3′ and downstream 5′-GTCTGTTGTTGCTTTTCAGC-3′ for rat CXCL12 gene; upstream 5′-ATGAACGCCAAGGTCGTGGT-3′ and downstream 5′-GGGCACAGTTTGGAGTGTTG-3′ for human CXCL12 gene; upstream 5′-CTGACTGGTACTTTGGGAAA-3′ and downstream 5′-GGAACACCACCATCCACAGG-3′ for rat CXCR4 gene; upstream 5′-CTGGTGGACATTGTGAAAGG-3′ and downstream 5′-TCTGCCTTCTGCTCAGTTTC-3′ for rat PARP-1 gene; upstream 5′-GCCAGCCGAGATCGTGGAAC-3′ and downstream 5′-GATCATGGGCGGGTGGTGGT-3′ for rat YY1 gene; upstream 5′-AGATTACTGCCCTGGCTCCT-3′ and downstream 5′-ACATCTGCTGGAAGGTGGAC-3′ for rat β-actin gene. The real-time PCR program for quantitative RT-PCR was comprised of an initial step at 50°C for 2 min, followed by an initial denaturation step at 95°C for 10 min and a subsequent 2-step PCR program at 95°C for 15 s and 60°C for 60 s for 40 cycles. Negative controls lacking the template were used in all RT-qPCR reactions. The expression levels of target genes were related to the averaged expression level of rat β-actin as the housekeeping gene.

### Statistical Analysis

The results are expressed as means ± SEM of triplicate data. Student’s *t* test was used to determine the significance of differences between two groups; *p*<0.05 was considered significant.

## Results

### Increased Presence of CXCL12 Improves Pancreatic Beta Cell Survival during Oxidative Stress Induced by a Diabetogenic Stimulus

**Figure 1:**
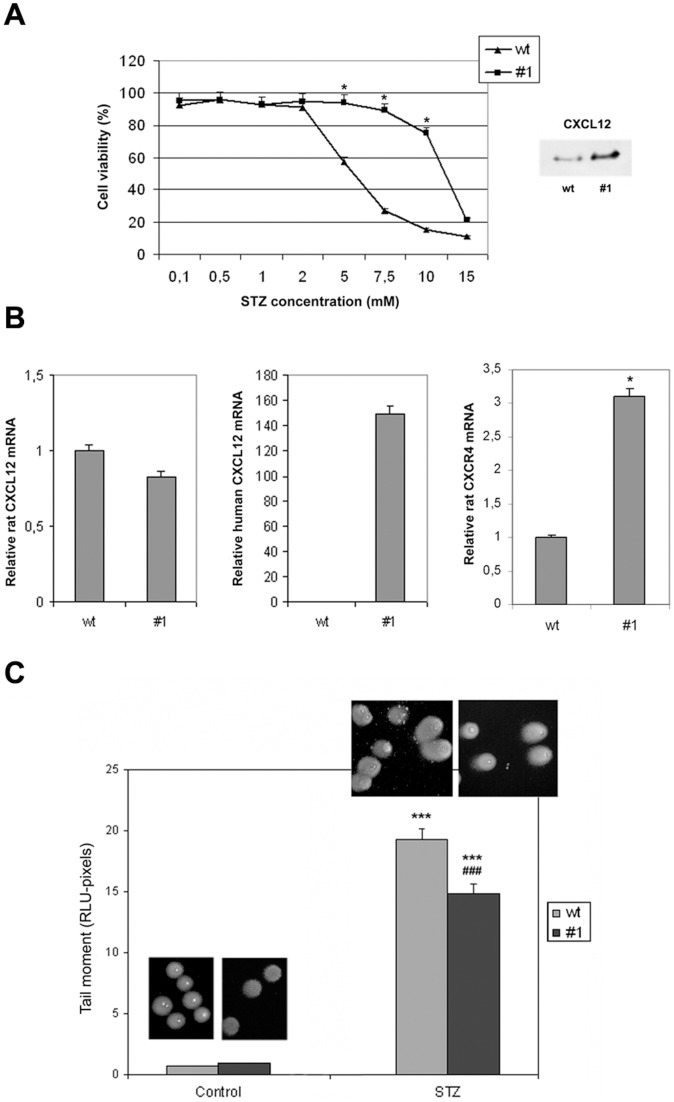
Overexpressed CXCL12 promotes better survival of injured pancreatic beta cells. (A) Viability assay performed on wt and clone #1 cells after treatment with increasing STZ concentrations; mean values for clone #1 were significantly different (*) from those for wt cells treated with the same STZ concentration (p<0.05). Increased presence of CXCL12 protein in the cell culture medium was verified by immunoblot analysis with anti-CXCL12 antibody (figure inset): lane 1– wt cells; lane 2– clone #1 cells. (B) Relative mRNA levels determined by real-time PCR and presented as ratios of *ratCxcl12/β-actin, humanCXCL12/β-actin* and *ratCxcr4/β-actin.* Mean values of clone #1 were significantly different (*) from those of wt cells (p<0.05). (C) Assessment of DNA damage by the Comet assay in wt cells and clone #1 after STZ treatment. The mean values of the tail moment (the parameter of DNA damage), of STZ-treated cells were significantly different (*) from those of untreated control cells (p<0.05); the mean values of the tail moment of the STZ-treated clone #1 cells were significantly different (#) from those of STZ-treated wt cells (p<0.05). All results are expressed as the means±SEM from three separate experiments performed in triplicate.

To confirm the prosurvival potential of CXCL12, the RIN-5F rat pancreatic beta cell line (wt) and its counterpart possessing a stably integrated human gene for CXCL12 (clone #1) were exposed to increasing concentrations of STZ (Fig. 1A). Monitoring cell viability revealed that increasing STZ concentrations were toxic to proportionally more wt than clone #1 cells. STZ concentration of 5 mM induced death in about 42% and 6% of wt and clone #1 cells, respectively. This STZ concentration was taken as EC_50_ in all further experiments. The viability data indicates that the overexpressed CXCL12 gene and increased presence of CXCL12 protein in the cell culture medium (inset in Fig. 1A) exerted a prosurvival effect on pancreatic beta cells through autocrine and paracrine signalling. RT-qPCR analysis of the rat and human genes encoding for CXCL12 revealed no significant differences in rat *Cxcl12* expression between wt and clone #1 cells while high expression of human *Cxcl12* was observed in clone #1 cells, confirming its stable genomic integration (Fig. 1B). The existence of paracrine and autocrine signalling was confirmed by the high expression of *Cxcr4* in clone #1 compared to wt cells (Fig. 1B).

The Comet assay was used to assess the levels of STZ-induced DNA damage (Fig. 1C). A considerable percentage (90–95%) of wt and clone #1 cells was without DNA tails (Fig. 1C) which reflects the absence of DNA breaks. A 30 min treatment with 5 mM STZ was accompanied by increasing amounts of DNA in the Comet tails due to increased DNA damage. Estimation of the tail moments confirmed that DNA damage was significantly less pronounced in clone #1 than in wt cells.

### Characterization of the Cxcl12 Gene Promoter Region

**Figure 2:**
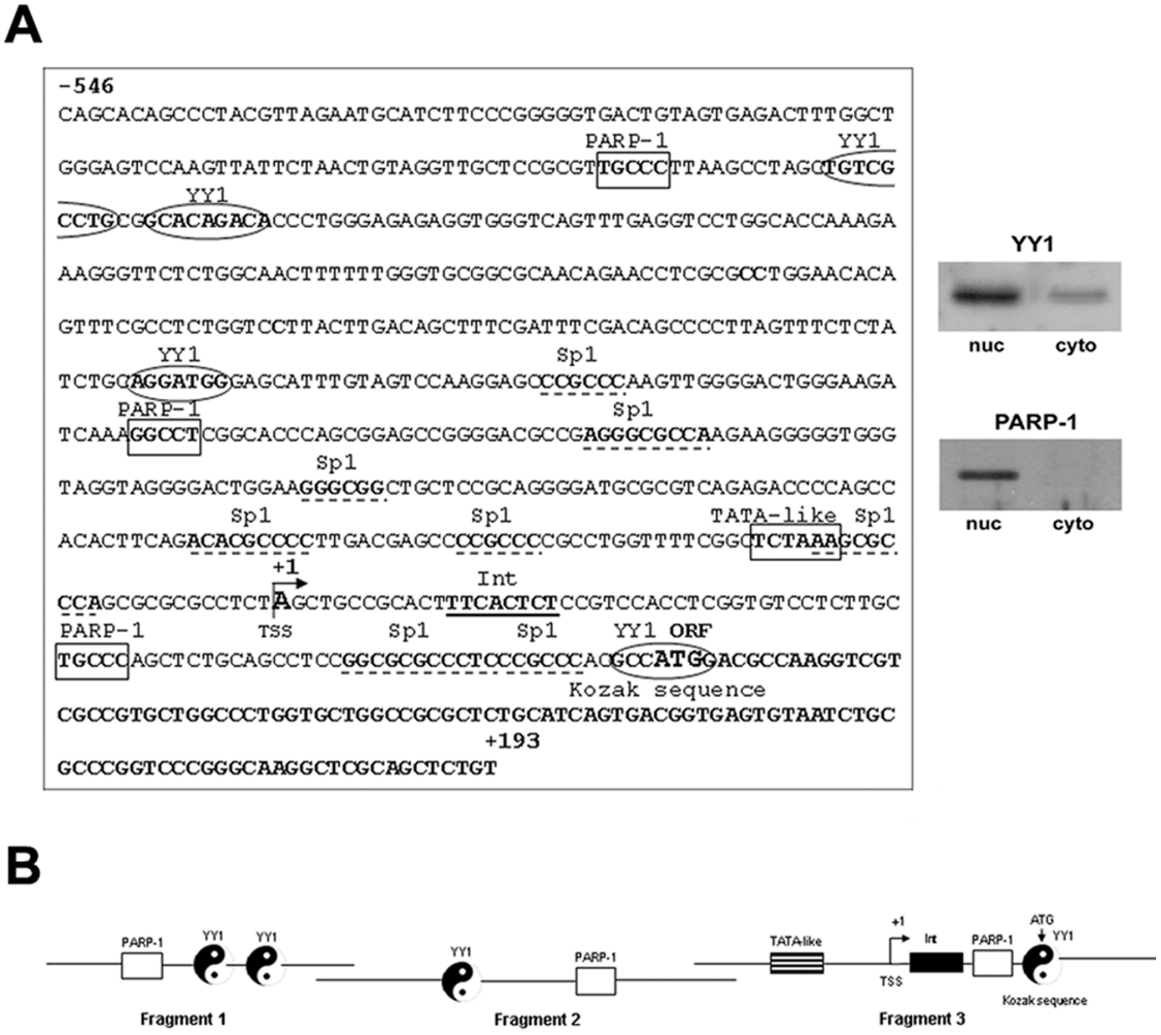
Analysis of the 739 bp *Cxcl12* promoter for transcription factor binding sites. (A) Putative YY1 sites are enclosed in an oval; Sp1 binding sites are underlined; three identified published PARP-1 DNA binding motifs [17], [22] are marked by rectangles; the TATA-like and Int elements and Kozak sequence are indicated. Expression of YY1 and PARP-1 in the Rin-5F cell line was verified by immunoblot analysis (figure inset). (B) A schematic diagram of the three promoter fragments used in ChIP analysis; each promoter fragment contained at least one putative PARP-1 and YY1 motif, represented by a rectangle and yin-yang symbol, respectively.

To elucidate the molecular mechanisms that regulate *Cxcl12* transcription in pancreatic beta cells, we defined the *Cxcl12* promoter sequence by computer analysis (Genomatix, Munich). The Genomatix prediction of the *Cxcl12* promoter revealed a 739 bp long sequence. The prediction covered sequences between positions 21520023 and 21520762 (NCBI Reference Sequence: NC_005103.3). The predicted *Cxcl12* promoter possesses a non-canonical TATA box (from −25 to −20 bp) with a cytosine instead of adenosine at the second position of the TATA box, as reported [39]. It also contains an initiator element (Int; from +13 to +20 bp), in agreement with the Int-consensus (KYAY*TCYYY) surrounding the secondary transcription start site. Downstream from the Int element lies a putative Kozak sequence (GCCATGG) containing the initiation ATG codon (Fig. 2A), consistent with its vertebrate consensus (RCCATGG) [40].

Further, the *Cxcl12* promoter was analysed for potential YY1 binding sites. Transcription factor-binding site analysis identified four putative YY1 binding sites (Fig. 2A, B) at positions −432/−424 bp, −421/−413 bp, −245/−239 bp, and +84/+90 bp. Binding sites for Sp1, a trans-regulator of the human *Cxcl12* gene, were confirmed. PARP-1 binding sites were also defined (Fig. 2A, B) according to the published literature data: 5′-GGCCT-3′ (−187/−183 bp), we predicted previously using *cis*-diammine-dichloro-platinum II cross-linking procedure [17] and 5′-TGCCC-3′ (at positions: −448/−444 bp and +45/+49 bp) taken from Akiyama *et al.* [22]. The both proteins are expressed in used Rin-5F cell line (inset in Fig. 2A).

### PARP-1 and YY1 are Part of the Transcription Machinery that Regulates Cxcl12 Expression in vitro and in vivo

**Figure 3:**
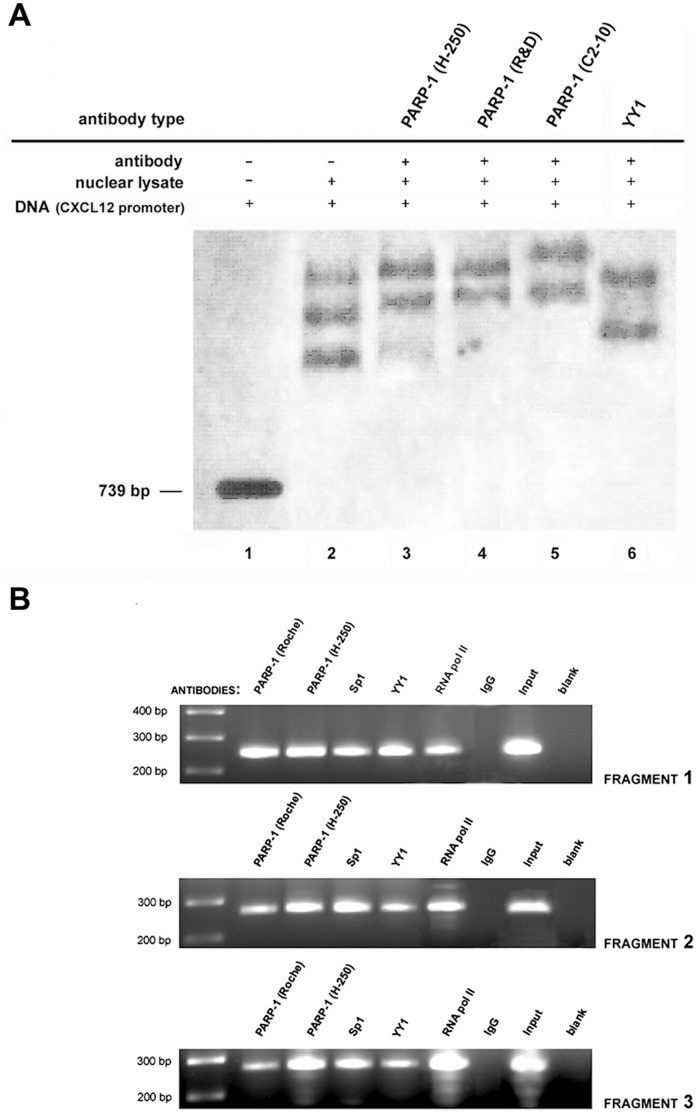
PARP-1 and YY1 binding affinity toward the *Cxcl12* promoter. (A) EMSA was performed with end-protected (biotinylated) fragments to permit PARP-1 binding exclusively to DNA-internal motifs. Super-shift analysis was performed using anti-PARP-1 and anti-YY1 antibodies as indicated. (B) ChIP analysis was performed with PARP-1 and YY1 antibodies. PARP-1 and YY1 binding was verified for each *Cxcl12* promoter fragment; fragment 1 is 246 bp; fragment 2 is 265 bp; fragment 3 is 268 bp. For immunoprecipitation, RNA pol II served as a positive, and IgG as a negative control. In the PCR reaction the positive control was genomic DNA (input); a water-only (blank) was the negative control.

PARP-1 and YY1 binding affinity toward the *Cxcl12* promoter was examined by EMSA. Several nucleoprotein complexes were formed between the *Cxcl12* promoter and nuclear proteins isolated from wt cells (Fig. 3A; lane 2). Super-shift analysis with different anti-PARP-1 and anti-YY1 antibodies revealed that PARP-1 (Fig. 3A; lanes 3, 4, 5) and YY1 (lane 6) proteins were present in the nucleoprotein complexes.

ChIP analysis was employed to determine whether the protein-DNA interactions at the *Cxcl12* promoter detected by EMSA also occur *in vivo* (Fig. 3B). To examine protein binding to the promoter at a higher resolution, the 739 bp promoter was divided into 3 fragments (Fig. 2B): fragment 1 (−546 to −299 bp); fragment 2 (−319 to −54 bp); fragment 3 (−74 to +193 bp). PARP-1 and YY1 binding to all three *Cxcl12* promoter fragments was observed (Fig. 3B). Each promoter fragment contains at least one PARP-1 and YY1 binding site (Fig. 2A, B). The putative Kozak sequence in the *Cxcl12* promoter overlaps with the YY1 binding motif in the third promoter fragment, indicating that the third fragment contains the translation start site. The chromatin-associated Sp1 transcription factor, analysed as a positive *Cxcl12* promoter-binding protein [39], was also found at the *Cxcl12* promoter (Fig. 3B; lane 4) which correlates with its multiple binding sites within Cxcl12 promoter (Fig. 2A).

### PARP-1 is an Inhibitor and YY1 a Strong Activator of CXCL12 Gene Transcription

**Figure 4:**
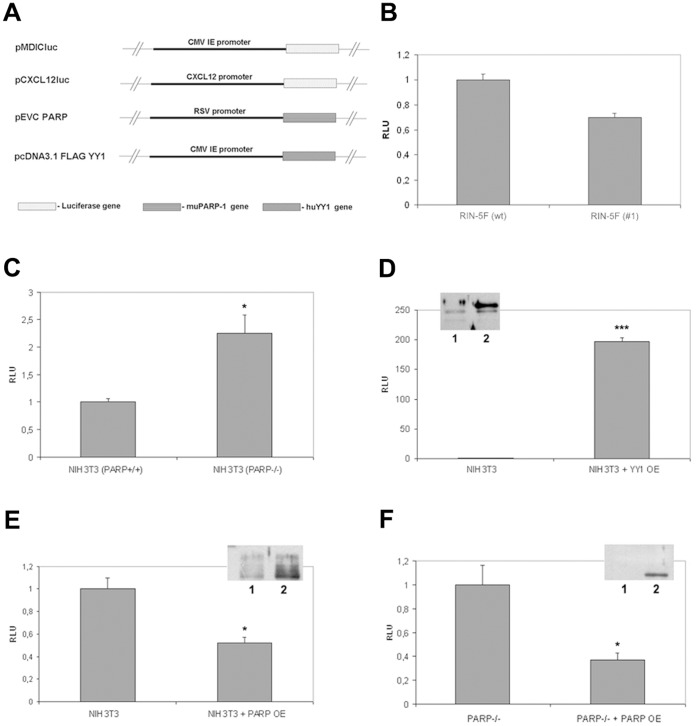
PARP-1 downregulates and YY1 upregulates *Cxcl12* promoter activity. (A) Constructs used in transfection experiments: pMDICluc – control plasmid; the luciferase gene was driven by the CMV promoter; pCXCL12luc – reporter construct; the luciferase gene under the control of the *Cxcl12* promoter; pECV PARP – PARP-1 cDNA expression construct; pcDNA3.1FLAGYY1– expression vector containing a YY1 expression unit. pMDICluc and pCXCL12luc constructs were used for transfection of (B) Rin-5F wt and clone #1 cells and (C) NIH3T3 (PARP^+/+^) and NIH3T3 (PARP^−/−^) mouse embryonic fibroblasts. Activity of the *Cxcl12* promoter was expressed relative to the activity of the control CMV promoter. (D) Transfection of NIH3T3 cells with pCXCL12luc and combined pCXCL12luc/pcDNA3.1FLAGYY1 constructs. Overexpression (OE) of YY1 was confirmed by immunoblot analysis with anti-YY1 antibody (figure inset): lane 1– NIH3T3 cell lysate; lane 2– NIH3T3 cell lysate after pcDNA3.1FLAGYY1 transfection. Transfection with pCXCL12luc or with the combination of pCXCL12luc/pECV PARP was performed in (E) NIH3T3 (PARP^+/+^) cells and (F) NIH3T3 (PARP^−/−^) cells. PARP-1 overexpression (OE) was verified by immunoblot analysis (figure insets): lane 1– NIH3T3 cell lysate; lane 2– NIH3T3 cell lysate after pECV PARP transfection. Statistical significance (*) p<0.05. All results are expressed as the means±SEM, obtained from three separate experiments performed in triplicate.

To analyse *Cxcl12* promoter activity, the promoter was cloned into a luciferase expression vector (pCXCL12luc) (Fig. 4A). Transfection of wt and clone #1 cells with pCXCL12luc showed slight decrease in *Cxcl12* promoter activity in clone #1 cells. However, this reduction in promoter activity was not statistically significant, indicating that *Cxcl12* does not influence its own expression (Fig. 4B). To examine the effect of PARP-1 on *Cxcl12* transcription, promoter activity was examined in NIH3T3 fibroblasts (PARP^+/+^) and PARP-1 knockout fibroblasts (PARP^−/−^) (Fig. 4C). Activity of the *Cxcl12* promoter was expressed relative to the activity of the control CMV promoter. Functional analysis using the luciferase assay showed significantly enhanced (2-fold) *Cxcl12* promoter activity in PARP^−/−^ compared to PARP^+/+^ fibroblasts (Fig. 4C). This was confirmed when a PARP-1 expression construct (pEVC PARP) was introduced into PARP^+/+^ and PARP^−/−^ cells which caused reduced expression of pCXCL121luc (Fig. 4E, F, respectively). These results indicate that PARP-1 downregulates *Cxcl12* promoter activity.

To explore the influence of YY1, NIH3T3 fibroblasts were transfected with pCXCL12luc and cotransfected with the pcDNA3.1FLAGYY1 expression vector containing a YY1 expression unit (Fig. 4D). *Cxcl12* promoter activity was enhanced 196-fold when YY1 was overexpressed. This result suggests that YY1 strongly upregulates *Cxcl12* promoter activity. YY1 (inset to Fig. 4D) and PARP-1 (inset to Fig. 4E, F) overexpression was verified by immunoblot analysis of cell lysates.

### STZ-induced Toxicity in Pancreatic Beta Cells is Accompanied by Changed Regulation of Cxcl12 Promoter Activity

**Figure 5:**
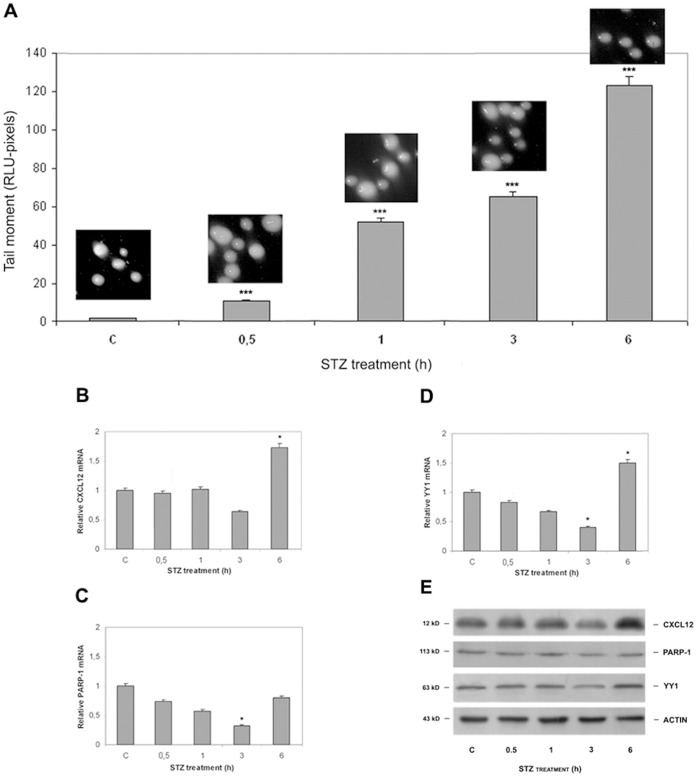
Increasing time of STZ treatment caused changes in *Cxcl12, Parp-1* and *Yy1* transcription and protein expression in Rin-5F wt cells. (A) DNA damage was determined by the Comet assay (the tail moment was the parameter of DNA damage). Transcription of *Cxcl12* (B), *Parp-1* (C) and *Yy1* (D) after increasing times of exposure to STZ was estimated by RT-qPCR. Relative mRNA levels are presented as the ratios of *Cxcl12/β-actin*, *Parp-1*/*β-actin* and *Yy1/β-actin.* (*) Mean values were significantly different from those of untreated control cells (p<0.05). Results are expressed as the means±SEM from three separate experiments performed in triplicate. (E) Immunoblot analysis was performed with anti-CXCL12, anti-PARP-1, anti-YY1 and anti-β-actin (loading control) antibodies using cell lysates isolated from control and STZ treated cells at defined time points.

During the early stage of oxidative stress, i.e. after a 30 min treatment of wt cells with 5 mM STZ (EC_50_), the mRNA levels for *Cxcl12*, *Parp-1* and *Yy1* decreased slightly (but not significantly) below the respective basal mRNA values (Fig. 5B, C, D; bar 2). To analyse the later stages of oxidative stress, wt cells were treated with 5 mM STZ for increasing times (1, 3, and 6 h). DNA damage was monitored by the Comet assay (Fig. 5A). The level of *Cxcl12* transcription was estimated using RT-qPCR (Fig. 5B). As can be seen in Fig. 5A, DNA damage increased with the length of exposure of cells to STZ. Estimation of the amount of transcribed *Cxcl12* at the respective time points confirmed that exposure to 5 mM STZ for 6 h caused a peak in *Cxcl12* mRNA synthesis (Fig. 5B; column 5), suggesting that when DNA is extensively damaged (Fig. 5A; column 5), *Cxcl12* transcription is induced. The extended STZ treatment was accompanied by similar patterns of increased *Yy1* and *Parp-1* mRNAs (Fig. 5C, D). The *Parp-1* mRNA level increased slightly after 6 h of STZ exposure, but remained below the control value (Fig. 5C; column 5), while the *Yy1* mRNA level was significantly above the control level (Fig. 5D; column 5), suggesting that long-term exposure of pancreatic beta cells to STZ-induced diabetogenic stimulation was accompanied by increased *Yy1* transcription. The mRNA levels estimated by RT-qPCR were accompanied with analysis of the protein levels at the same time points using immunoblot analysis (Fig. 5E). Increased amount of YY1, due to its ability to upregulate *Cxcl12* transcription, may be responsible for higher *Cxcl12* expression also observed after 6 h of STZ treatment.

**Figure 6:**
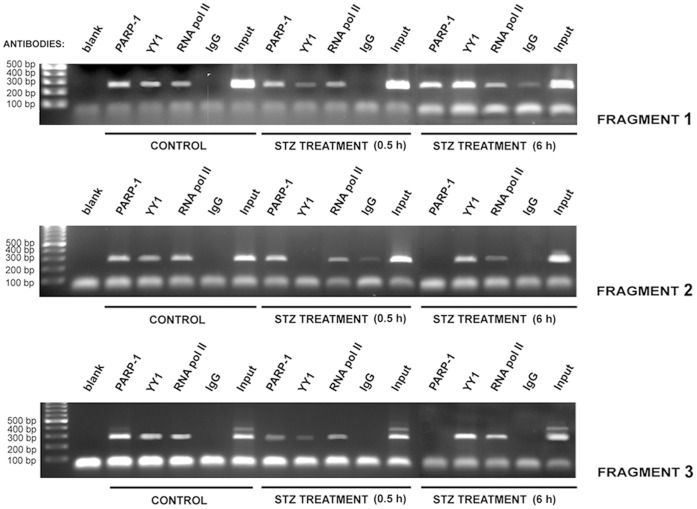
STZ-induced changes in *Cxcl12* promoter regulation. ChIP analysis was used to investigate PARP-1 and YY1 binding affinity toward the *Cxcl12* promoter during the early (0.5 h) and late stage of oxidative stress (6 h). Immunoprecipitation was performed with anti-PARP-1 and anti-YY1 antibodies. The controls in immunoprecipitation and PCR are shown in Fig. 3.

PARP-1 and YY1 binding to the *Cxcl12* promoter after short- and long-term cell exposure to STZ was examined by the ChIP assay. Chromatin was isolated from Rin-5F wt control cells and cells exposed to 5 mM STZ for 30 min (early stage of oxidative stress) and 6 h (later stage of oxidative stress) (Fig. 6). The ChIP experiment revealed that the STZ treatment for 30 min lowered PARP-1 affinity for fragment 3 while its affinity for *Cxcl12* fragments 1 and 2 remained as in the control. As a result of the 30 min exposure to STZ, YY1 exhibited very low binding to fragments 1 and 3 and no binding to fragment 2 (Fig. 6). The STZ treatment for 6 h revealed opposite binding pattern for PARP-1 and YY1 if compare to 30 min STZ treatment. PARP-1 was bound to *Cxcl12* promoter fragment 1 but displayed no binding affinity to promoter fragments 2 and 3 (Fig. 6). As expected, YY1 displayed increased binding affinity for all three *Cxcl12* promoter fragments, which resulted in the increased CXCL12 gene expression during extended (6 h) pancreatic beta cell injury.

### Influence of PARP-1 Inhibition on Cxcl12 Promoter Regulation

**Figure 7:**
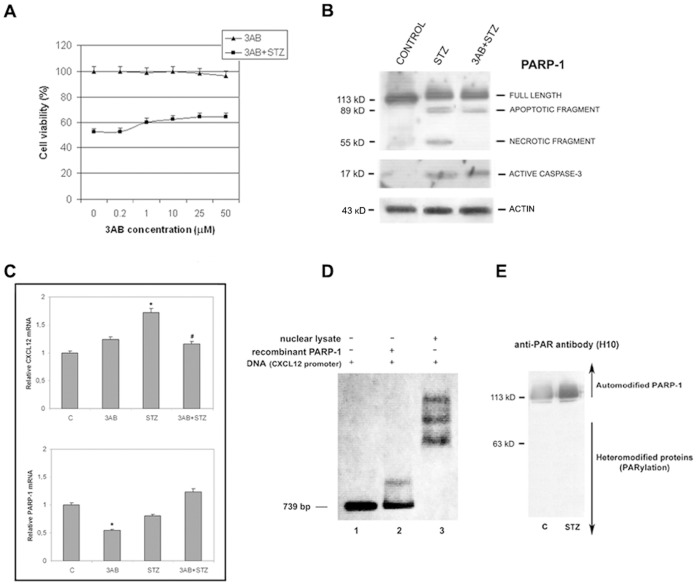
The effect of PARP-1 inhibition on *Cxcl12* promoter regulation. (A) Viability assay was performed with wt cells treated with increasing concentrations of 3AB, followed by STZ treatment. (B) Immunoblot analysis was performed with anti-PARP-1, anti-caspase 3 and anti-β-actin (loading control) antibodies using cell lysates isolated from control, STZ-treated and cells pre-treated with 3AB, followed by STZ treatment. The apoptotic (89 kD) and necrotic (55 kD) PARP-1 fragment are indicated. (C) *Cxcl12* and *Parp-1* transcription after treatments with either 3AB or STZ, and after incubation with 3AB, followed by STZ treatment, was estimated by RT-qPCR. Relative mRNA levels are presented as the ratios of *Cxcl12/β-actin* and *Parp-1*/*β-actin.* (*) Mean values were significantly different from those of untreated control cells (p<0.05). (^#^) Mean values were significantly different from those of STZ-treated cells (p<0.05). Results are expressed as the means±SEM from three separate experiments performed in triplicate. (D) EMSA showing binding of recombinant PARP-1 and total nuclear proteins to the *Cxcl12* promoter (lanes 2 and 3, respectively). (E) Nuclear proteins from control (C) and STZ-treated wt cells (STZ) probed with anti-ADP-ribose antibody to detect automodified PARP-1 and other ADP-ribosylated proteins.

PARP-1 enzymatic activity was chemically inhibited using 3AB, a general PARP-1 enzymatic activity blocker. Treatment of wt cells with 50 µM 3AB had no apparent effect on cell viability, while in the cells treated with 5 mM STZ we observed slight improvement in cell survival (Fig. 7A). When wt cells were treated with STZ together with 3AB, the disappearance of the necrotic PARP-1 fragment indicates that the necrotic pathway was turned off as a consequence of the inhibition of PARP-1 enzymatic activity (Fig. 7B). Besides pronounced PARP-1 apoptotic fragment, PARP-1 inhibition was accompanied with the activation of the main effector caspase 3 (Fig. 7B).

RT-qPCR analysis did not reveal significant changes in PARP-1 gene transcription in the presence of STZ, either alone or together with 3AB. *Cxcl12* mRNA levels exhibited pronounced differences (Fig. 7C). The treatment with STZ led to upregulation of *Cxcl12* while treatment with STZ and 3AB downregulated *Cxcl12* transcription (Fig. 7C). We suggest that intensive binding of non-automodified PARP-1 to the *Cxcl12* promoter partially suppressed promoter activity. The ability of non-automodified PARP-1 to bind the *Cxcl12* promoter was proven in an EMSA experiment using recombinant (non-automodified) PARP-1 (Fig. 7D; lane 2). In addition, we observed that YY1 was not ADP-ribosylated, in both physiological and STZ-compromised conditions (Fig. 7E) that allows its binding to *Cxcl12* promoter and subsequent induction of the *Cxcl12* expression in the later stage of oxidative stress.

## Discussion

The positive impact of the elevated expression of the chemokine CXCL12 on prosurvival and proliferative phenotype in pancreatic islet cells has been observed recently [5]. Based on our experimental results and information from other studies [5], [6], [7], we confirmed importance of CXCL12 as a pancreatic beta cell prosurvival factor. Going a step further, we clarified the role of PARP-1 and YY1 in the regulation of the *Cxcl12* transcription, suggesting that transcriptional activation of the *Cxcl12* promoter certainly depends on the finely balanced functional interplay of these proteins.

For the first time we defined and analysed the promoter sequence of the rat CXCL12 gene using computer analyses (Genomatix, Munich). Up to now, transcriptional regulation of the *Cxcl12* promoter has been described in human [39], [41], [42], [43], [44] and mouse [45], [46] cells. Their results on mammalian *Cxcl12* promoter characterization and our data analysis show that Cxcl12 promoter contains a non-canonical TATA box and a downstream Int element for the initiation of transcription [39], [45] that can function as an alternative promoter in eukaryotic genes that lack the classical TATA box [47]. In the rat *Cxcl12* promoter, Int is located 31 bp downstream from the non-canonical TATA box, while in the human this distance is 26 bp [39]. The Kozak sequence (CCATGG) with the contained ATG initiation codon was designated as a translation start site [40]. In human and mouse *Cxcl12* promoters, Kozak sequence is not particularly defined so far [39], [40]. We observed the high degree of similarity between human and rodent Kozak sequence, although the Kozak sequence is not strictly conserved in eukaryotic mRNAs [48]. As in the mouse *Parp-1* promoter [17] we also observed overlap of the YY1 core-binding motif (ATG) with the Kozak sequence (GCCATGG) in the rat *Cxcl12* promoter. This is in agreement with the colocalization of the YY1 motif and the translation start site in many human promoters [28]. Our analysis of the organization of the rat *Cxcl12* promoter also revealed the presence of GC-rich sequences in the 5'-flanking region, as reported previously [39]. Ubiquitous expression of the *Cxcl12*, except in blood cells, is consistent with the presence of the GC-rich sequence in the 5'-flanking region and the non-canonical TATA box, common features of housekeeping genes, as we also observed for the mouse PARP-1 gene [17].

Up to now several transcription factors involved in the regulation of CXCL12 gene transcription have been described in human and mouse. The transcription factors of the early B cell factor (EBF) family [45], Sp1 [39], STAT3 [46], c-myb [43], C/EBPβ [41], [42] and Ets-related molecule [49] were defined as promoter activators while transcription factors Foxf1 [50] and p53 [51] were identified as a promoter suppressors. The 1 kb long proximal human *Cxcl12* promoter possesses 15 putative Sp1 binding motifs, of which 6 are important for basal human *Cxcl12* expression [39]. Our computer analysis confirmed the presence of 8 putative Sp1 binding sites within the *Cxcl12* promoter, all surrounding the non-conventional TATA box. In our study, transcription factor-binding site analysis showed several putative binding sites within the *Cxcl12* promoter for the following transcription factors: C/EBPβ, C/EBPα, FOXO3a, HMGI/Y, p53, STAT3 and NF-κB. EMSA experiments and ChIP assays confirmed binding affinities for some of these transcription factors that will be published elsewhere. Although PARP-1 has been characterized as a transcription factor only recently, an increasing number of reports have been published, indicating that PARP-1 can also bind to DNA in a sequence-specific manner. While several PARP-1 binding motifs have been published (reviewed in [17], [18], [21], [22], [23]), a DNA binding consensus sequence for PARP-1 is still not available in the existing computer analysis tools. For that reasons we analysed the previously published PARP-1 DNA binding sequences [17], [22].

Our transfection experiments clearly show that PARP-1 has a pivotal role in partial suppression of the *Cxcl12* promoter, allowing for its constitutive expression. During the oxidative stress, due to PARP-1 automodification and the negative charge of ADP-ribose polymers, PARP-1 is detached from the *Cxcl12* promoter thus allowing for enhanced *Cxcl12* transcription. This momentum of PARP-1 release from the *Cxcl12* promoter allows for increased gene transcription and places PARP-1 in the position of a weak suppressor, enabling basal promoter activity. The suppressive effect of PARP-1 on gene transcription was observed for the Tracp gene in pre-osteoclastic cells [52] and for its own gene in mouse fibroblasts [17]. Our observation is in agreement with Amiri *et al.* [53] who revealed the suppressive effect of PARP-1 on CXCL1 gene expression. In contrast, Nirodi *et al*. [21] reported that PARP-1 may act as a coactivator of CXCL1 gene transcription. Besides *Cxcl12*, PARP-1 is also involved in the regulation of several other diabetes-related genes. PARP-1 acts as a corepressor for the Foxo1 gene, which could play an important role in proper cell proliferation and in the response to oxidative stress [54]. Furthermore, Akiyama *et al.* [22] demonstrated that PARP-1 forms the active complex for *Reg* transcription with some nuclear proteins, and that complex formation was stabilized when PARP-1 was not automodified.

The CXCL12 gene was initially considered to be constitutively expressed. However, it was recently established that its transcription is induced by cell injury [6], in response to cytokines and cell confluence [41] and during cell growth arrest and hypoxia [55]. Recently, Liu *et al.* [6] observed short-term influence of exogenous CXCL12 on the induction of its own gene expression. In our experiments, long-term (chronic) presence of CXCL12 in clone #1 cells has no autoregulatory transcriptional potential. Under conditions of YY1 overexpression, which is followed by its increased binding to *Cxcl12* promoter, we observed highly increased *Cxcl12* promoter activity. This lends further support to the concept that the *Cxcl12* promoter is inducible, i.e., characterized by a low basal activity that greatly increases upon induction. Baumeister *et al.* [56] reported similar pattern of transcriptional regulation by YY1 concerning Grp78 gene promoter regulation. The authors proposed that YY1 has no effect on *Grp78* promoter basal activity, however in cells undergoing ER stress YY1 strongly enhances *Grp78* promoter induction. The observation that both YY1 [35] and CXCL12 genes [55] are upregulated by hypoxia points to the interrelatedness of their responses to stress signals. In contrast to its role in *Cxcl12* transcription, YY1 represses the activities of *Cxcr4* and *Cxcr7* promoters through its binding to upstream regions on their promoters [35]. As a potential element of a negative feed-back mechanism controlling the CXCL12/CXCR4 axis, this action represents an additional level of control of *Cxcl12* expression by YY1. Therefore, we believe that YY1 could be a major regulator which helps beta cells to transcribe important proteins that help coping with oxidative stress. In addition, strong transcriptional induction of the prosurvival chemokine could be the pancreatic cell’s response to severe oxidative stress.

**Figure 8:**
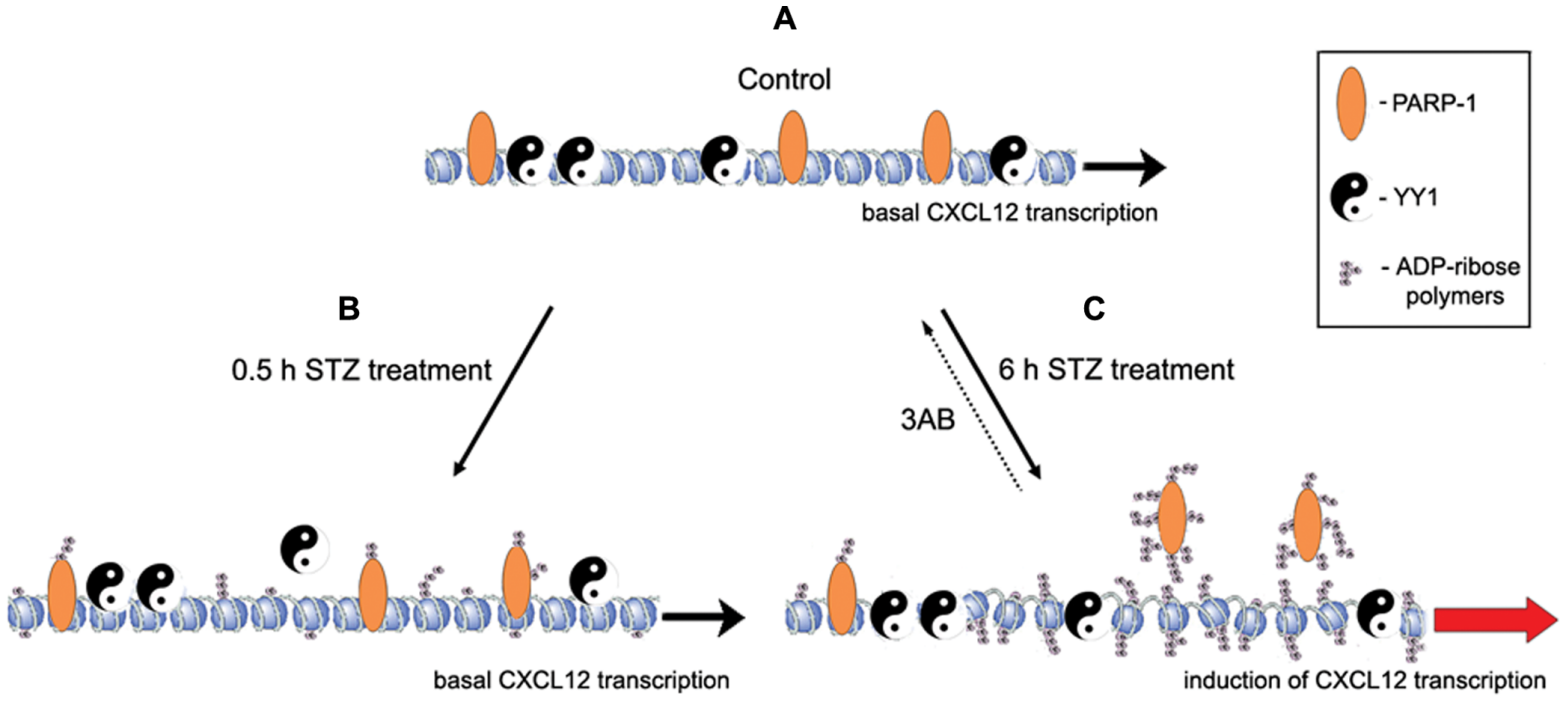
Regulation of *Cxcl12* promoter activity in pancreatic beta cells: a working model. (A) In the basal state, non-automodified PARP-1 (acting as a transcriptional inhibitor) and YY1 (acting as a transcriptional activator) strongly bind to the *Cxcl12* promoter, enabling its transcription (Fig. 3, 4). (B) DNA damage induced by STZ (diabetogenic-like) treatment causes PARP-1 binding to the DNA breaks, leading to a net increase in PARP-1 activity and consequently to poly(ADP-ribosyl)ation of PARP-1 and associated chromatin proteins. After 30 min of STZ treatment, DNA is moderately damaged (Fig. 5A), which results in weak poly(ADP-ribosyl)ation so that most of PARP-1 molecules remain attached to the promoter, however, as YY1 partially dissociates from the promoter (Fig. 6), *Cxcl12* transcription is still at the basal level (Fig. 5B). (C) After 6 h of STZ treatment, the DNA is severely damaged (Fig. 5A), causing intensive poly(ADP-ribosyl)ation of both PARP-1 and associated chromatin proteins and PARP-1 dissociation from the promoter, with resulting opening of the chromatin. Severe beta cell injury induces YY1 expression (Fig. 5D, E). Furthermore, the open chromatin structure enables intense YY1 binding to the promoter (Fig. 6), upregulation of *Cxcl12* expression (Fig. 5B, E) and consequent increased cell survival. Treatment with PARP-1 inhibitor (3AB) causes reduced poly(ADP-ribosyl)ation and intense PARP-1 binding to the promoter, resulting in decreased *Cxcl12* transcription (Fig. 7C).

We have summarized the apparently opposing effects of PARP-1 and YY1 on *Cxcl12* transcription in a model presented in Fig. 8. Our findings suggest that in the physiological state *Cxcl12* transcription is supported by the binding of non-automodified PARP-1 and YY1 to the *Cxcl12* promoter. We hypothesize that under basal conditions when PARP-1 activity in pancreatic beta cells is negligible, the bound non-automodified PARP-1 offsets the elevation in *Cxcl12* transcription that occurs in the presence of YY1 (Fig. 8A). In response to cytotoxic signals, as PARP-1 enzymatic activity rises, PARP-1 automodification progressively lowers PARP-1 affinity for the *Cxcl12* promoter, since PARP-1 automodification blocks the ability of PARP-1 to bind to DNA [57] (Fig. 8B). This has an overall permissive effect on *Cxcl12* transcription. However, short-lasting exposure to STZ-induced diabetogenic signals was accompanied by a decrease in YY1 binding to DNA and consequent removal of its stimulatory effect on *Cxcl12* expression (Fig. 8B). Prolonged exposure to cytotoxic signals was followed by significantly enhanced PARP-1 automodification and decreased binding affinity for *Cxcl12* promoter (Fig. 8C). In contrast, prolonged pancreatic beta cell injury is followed by increased YY1 binding to *Cxcl12* promoter that will cause upregulation of the CXCL12 gene expression (Fig. 8C). It would appear that at relatively low levels of cytotoxicity, an increase in *Cxcl12* transcription that would result from the lifting of transcriptional suppression by PARP-1, is tempered by the withdrawal of YY1 stimulation. In contrast, severe beta cell injury was associated with increased *Yy1* expression that was followed by increased binding of YY1 protein to the *Cxcl12* promoter. The promoter assumed a more relaxed conformation due to a major increase in PARP-1 activity with resulting ADP-ribosylation of chromatin proteins, and the complete lifting of transcriptional suppression as a result of extensive PARP-1 automodification. The sum effect of these events is the induction of *Cxcl12* expression and subsequent prosurvival actions of CXCL12.

### Concluding Remarks

Several transcription factors involved in beta cell functioning, differentiation, proliferation and survival have been identified so far (reviewed in [58]). Novel drugs, which enhance the expression of key transcription factors, as in the case of Pdx-1 [59], could restore beta cell functions in diabetic patients. We believe that the present study is particularly relevant since two transcription factors that have been identified as important regulators of *Cxcl12* transcription could in the future serve as focal points for targeting *Cxcl12* expression. An open challenge is to find a way of inducing and/or suppressing transcription factors that participate in the transcriptional regulation of genes that could improve beta cell functioning. This strategy could advance the treatment of diabetes.
